# MOG Antibody-Associated Disorders Following SARS-CoV-2 Vaccination: A Case Report and Literature Review

**DOI:** 10.3389/fneur.2022.845755

**Published:** 2022-03-01

**Authors:** Yuki Matsumoto, Ayane Ohyama, Takafumi Kubota, Kensuke Ikeda, Kimihiko Kaneko, Yoshiki Takai, Hitoshi Warita, Toshiyuki Takahashi, Tatsuro Misu, Masashi Aoki

**Affiliations:** ^1^Department of Neurology, Tohoku University Graduate School of Medicine, Sendai, Japan; ^2^Department of Neurology, Tohoku University Hospital, Sendai, Japan; ^3^Department of Neurology, National Hospital Organization Yonezawa Hospital, Yonezawa, Japan

**Keywords:** myelin oligodendrocyte glycoprotein (MOG), SARS-CoV-2, COVID-19, post-vaccination, mRNA vaccine, cerebellar peduncle

## Abstract

Myelin oligodendrocyte glycoprotein (MOG) antibody-associated disorder (MOGAD) is a newly identified autoimmune demyelinating disorder that is often associated with acute disseminated encephalomyelitis and usually occurs postinfection or postvaccination. Here we report a case of MOGAD after mRNA severe acute respiratory syndrome coronavirus 2 (SARS-CoV-2) vaccination. A previously healthy 68-year-old woman presented to our department with gradually worsening numbness on the right side of her face, which began 14 days after her second dose of an mRNA-1273 vaccination. The patient's brain MRI revealed a right cerebellar peduncle lesion with gadolinium enhancement, a typical finding of MOGAD. A neurological examination revealed paresthesia on her right V2 and V3 areas. Other neurological examinations were unremarkable. Laboratory workups were positive for serum MOG-IgG as assessed by live cell-based assays and the presence of oligoclonal bands in the cerebrospinal fluid (CSF). The patient's serum test results for cytoplasmic-antineutrophil cytoplasmic antibodies, perinuclear-cytoplasmic-antineutrophil cytoplasmic antibodies, GQ1b-antibodies, and aquaporin-4 antibodies (AQP4-IgG) were all negative. Tests for soluble interleukin (IL)-2 receptors in the serum, IL-6 in the CSF and skin pricks, and angiotensin converting enzyme tests were all unremarkable. The patient was diagnosed with MOGAD after receiving an mRNA SARS-CoV-2 vaccination. After two courses of intravenous methylprednisolone treatment, the patient's symptoms improved and her cerebellar peduncle lesion shrunk slightly without gadolinium enhancement. To date, there have only been two cases of monophasic MOGAD following SARS-CoV-2 vaccination, including both the ChAdOx1 nCOV-19 and mRNA-1273 vaccines, and the prognosis is generally similar to other typical MOGAD cases. Although the appearance of MOG antibodies is relatively rare in post-COVID-19–vaccine demyelinating diseases, MOGAD should be considered in patients with central nervous system (CNS) demyelinating diseases after receiving a SARS-CoV-2 vaccine.

## Introduction

The emergence in December 2019 of a novel coronavirus, the severe acute respiratory syndrome coronavirus 2 (SARS-CoV-2), has had devastating global consequences. To overcome the unprecedented effects of the pandemic, there was a rapid global effort to develop several vaccines against SARS-CoV-2, which resulted in several safe and efficacious immunogenic vaccines including ChAdOx1 nCOV-19 and mRNA-1273 (1, 2). In particular, the ChAdOx1 nCOV-19 vaccine has shown an acceptable safety profile and has demonstrated a 62.1–90% efficacy reduction in COVID-19 infections in a clinical trial involving 23,848 participants (1). In this particular trial, 3 cases of transverse myelitis occurred, of which 1 case was determined to be possibly related to the vaccination as this case occurred 14 days after a ChAdOx1 nCoV-19 second booster vaccination. The remaining 2 cases were determined to be unlikely related to vaccinations as the first case occurred 10 days after the individual received a first vaccine dose and the individual had a preexisting unrecognized condition of multiple sclerosis (MS). The second case was determined not to be related to the vaccination as this individual received a placebo vaccine (1). The mRNA-1273 vaccine, which was developed by Moderna and the Vaccine Research Center at the National Institute of Allergy and Infectious Diseases (NIAID), has demonstrated tremendous clinical efficacy and safety against COVID-19 (2). The common adverse events of the mRNA-1273 vaccine are considered mild, which include transient headache, pain, muscle spasms, and myalgia (2). To date, only several cases of central nervous system (CNS) demyelinating disease occurring after mRNA vaccinations have been reported (3).

Myelin oligodendrocyte glycoprotein (MOG) antibody-associated disease (MOGAD) is a newly identified disease entity that was initially identified mainly in pediatric cases of acute disseminated encephalomyelitis (ADEM), usually in postvaccination or postinfectious circumstances (4, 5). So far, there have been 15 cases of ADEM and acute hemorrhagic leukoencephalitis (AHLE) after COVID-19 infections occurring mainly in adults. Of these 15 cases, there has only been 1 pediatric case involving a 13-month-old female patient with an MOG antibody (6). Furthermore, despite many cases of postvaccination ADEM after SARS-CoV-2 vaccination, MOG antibody–positive cases are rare (7). Here we describe a case of typical cerebellar peduncle lesion with an identified MOG-IgG occurring 2 weeks after an mRNA-1273 vaccination. We also analyzed MOGAD cases following SARS-CoV-2 vaccinations in a literature review.

## Case Description

**Figure 1:**
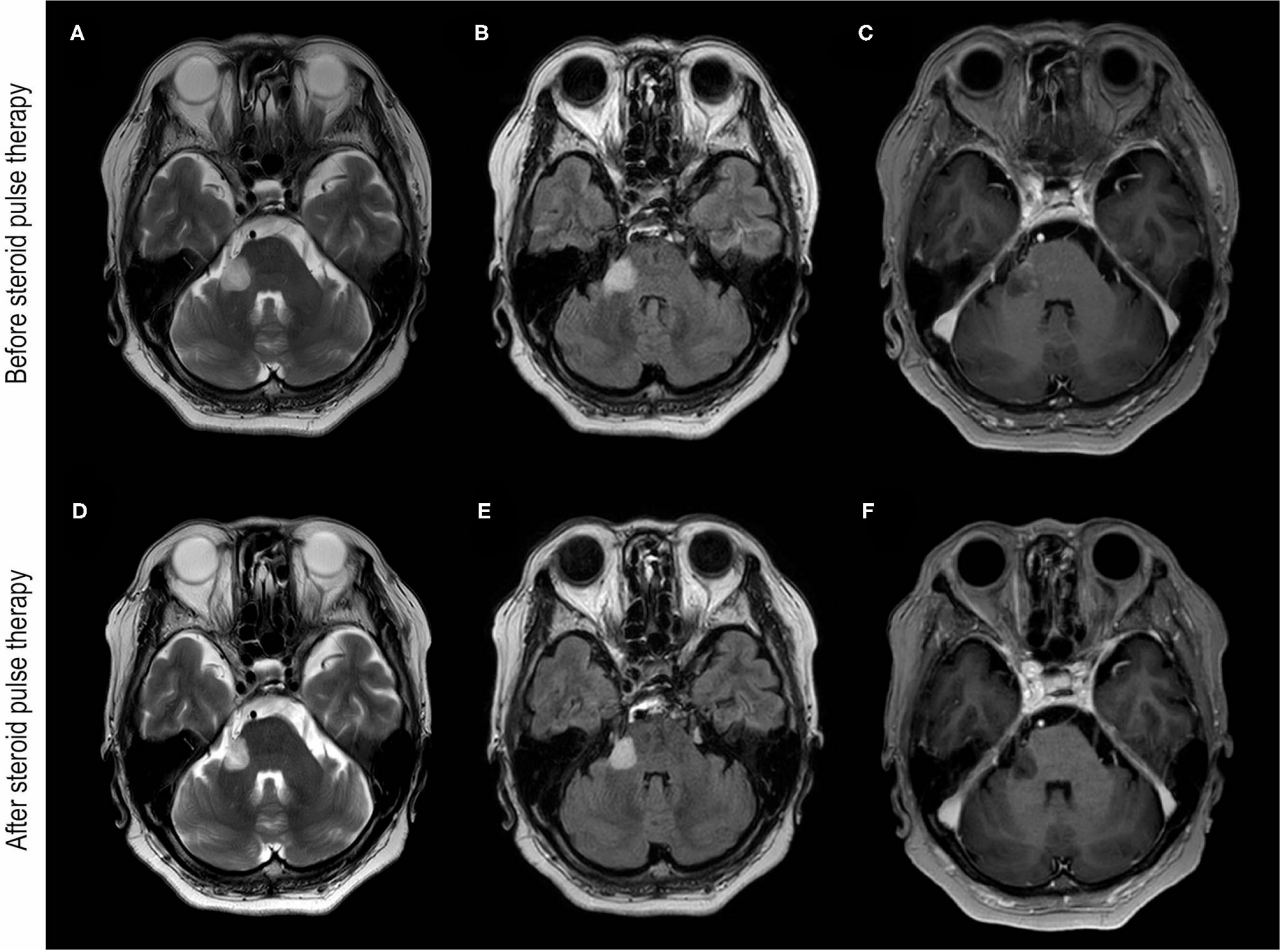
Brain MRI images before and after steroid pulse therapy. **(A–C)** Were obtained before steroid pulse therapy. Brain MRI of T2-weighted (A) and fluid-attenuated inversion recovery (FLAIR) **(B)** images showing hyperintensity of the cerebellar peduncle and the root of trigeminal nerve. **(C)** T1-weighted brain MRI with gadolinium enhancement (T1Gd) showing enhancement around the lesion. **(D–F)** were obtained after steroid pulse therapy. Brain MRI of T2-weighted **(D)** and FLAIR images **(E)** showing the reduction in the hyperintensity lesion of the cerebellar peduncle. **(F)** T1Gd brain MRI showing no gadolinium enhancement after steroid therapy.

A 68-year-old Japanese woman presented to our department with a complaint of gradually worsening numbness on the right side of her face 14 days after receiving a second dose of the mRNA-1273 vaccination. The patient developed a slight fever following the second dose of the mRNA-1273 vaccination, but the fever subsequently resolved. The patient had a remote history of well-controlled hypertension and had undergone the removal of the tail of her pancreas because of an intraductal papillary mucinous neoplasm 12 years before. The patient did not have a history of allergies or vaccine-induced side effects. There were no marked related findings in the patient's family's medical history. A neurological examination revealed paresthesia on the patient's right V2 and V3 areas. Other neurological examinations including pyramidal signs and ocular movements were normal. Blood tests showed no abnormal blood counts or abnormal biochemical profiles including those indicating the soluble interleukin (IL)-2 receptor. The patient's lumbar puncture revealed no pleocytosis or elevated proteins but did reveal positive oligoclonal bands. Her serum test results for the cytoplasmic-antineutrophil cytoplasmic antibody, perinuclear-cytoplasmic-antineutrophil cytoplasmic antibody, GQ1b-antibody, and aquaporin-4 IgG (AQP4-IgG) antibody were all negative. However, a live cell-based assay with titers of 1:512 using anti-IgG-Fc and 1:256 using anti-IgG1 as secondary antibodies (cut-off value; 1:128) was positive for MOG-IgG in the patient's serum, but was negative in her cerebrospinal fluid (CSF). Interferon-gamma release assays for tuberculosis were negative. An angiotensin-converting enzyme test, a tuberculosis skin test, and a chest X-ray were all unremarkable, and neurosarcoidosis was also deemed unlikely. A skin prick test was normal, and IL-6 in the CSF was not elevated. Neuro-Behcet's disease was also determined to be unlikely. A screening polymerase chain reaction (PCR) test of SARS-CoV-2 from nasopharyngeal swabs was negative. Brain MRI images showed a hyperintense lesion on the right lateral pontine, trigeminal nerve, and a middle cerebellar peduncle lesion was seen in T2-weighted and fluid-attenuated inversion recovery (FLAIR) images with a T1-gadolinium enhancement (Figure 1). The patient was diagnosed with MOGAD and administered 2 courses of intravenous methylprednisolone (IVMP), 1 g/d over 3 days. After two courses of IVMP, the cerebellar peduncle lesion shrunk slightly with a diminishment of the gadolinium enhancement (Figure 1). The paresthesia of the patient's face improved, but mild numbness on her right jaw remained at her discharge. The patient did not experience any relapse during the subsequent 6-month period without any treatment. Her MOG-IgG status in 6 months follow-up was positive for anti-IgG-Fc (1:128), but negative for IgG1 as secondary antibody.

We obtained written informed consent from the patient in the case presented. We were able to discover cases of MOGAD following COVID-19 vaccination by utilizing a PubMed search for terms including “COVID-19,” “vaccination,” and “myelin oligodendrocyte glycoprotein.” We also searched “COVID-19,” “vaccination,” and “neuromyelitis optica spectrum disorder” in order to ensure we had found all the documented cases of MOGAD.

## Discussion

We demonstrated a case of brainstem and cerebellar peduncle encephalitis with MOG antibodies developed after the patient's SARS-CoV-2 vaccination. In previous reports, we showed that middle cerebellar peduncle lesions are typical features of MOGAD, and therefore the lesion seen in the present case made a probable diagnosis of MOGAD (8). In contrast, MOGAD is less likely to occur in postvaccination CNS demyelinating diseases, because all cases of testing 10 MOG antibody cases were negative in recent systematic reviewed cases (7). It is possible to develop MOGAD after receiving the COVID-19 vaccination; MOGAD can develop from the postexposure production of pathogenic IgG via the molecular mimicry theory and vaccine-induced bystander inflammation-induced tissue injury and antigen presentation. In addition, the vaccination could simply unmask a potential preexisting autoimmune disease.

After the vaccination campaign during the “swine flu” in 1976, the number of Guillain-Barré syndrome (GBS) cases in the United States increased (9). Although the calculated relative risk was 6.2, an increase in the number of cases of multiple sclerosis was not observed (10). Later studies suggested that influenza vaccines can induce the production of antiganglioside GM1 antibodies in animal models by a molecular mimicry theory (11). It has been reported that the cumulative incidence of confirmed COVID-19 cases in MS patients is two-fold higher when the cases without PCR test in the Barcelona are included (12). However, a systematic review analysis has suggested that having MS does not significantly increase the mortality rate of COVID-19 (13). A pilot study, focused on the cross-reactivity between SARS-CoV-2 spike proteins and 50 different tissues using enzyme-linked immunosorbent assays, has indicated that SARS-CoV-2 spike proteins show the strongest immunoreactions with transglutaminases, myelin basic proteins, mitochondria, nuclear antigens, myosin, collagen, claudin5/6, and S100B (14). Furthermore, by analyzing the cross-reactions between host and microbial proteins using the Protein Data Bank, we found that SARS-CoV-2 proteins were found to possibly interact with many kinds of proteins including proteins involved in synaptic vesicle trafficking, endocytosis, axonal transport, neuronal transmission, thrombosis, inflammation, and the mitochondrial and blood brain barrier as well as protein growth factors (15). Although molecular mimicry between SARS-CoV-2 spike proteins and MOG is unclear, these cross-reaction theories show a potential correlation between CNS inflammation and demyelinating diseases including MOGAD and COVID-19.

In the current case report, the patient developed MOGAD 2 weeks after receiving an mRNA vaccination containing the SARS-CoV-2 spike protein, which is considered sufficient time for the creation of autoantibodies (16). Initially, the vaccinated antigen is taken up by dendritic cells, then trafficked to the draining lymph node, and interacted with activated T and B cells. T cell-dependent B cell maturation results in specific antibody production by plasma cells, which could induce a rapid rise in serum antibody levels over a subsequent 2-week period. Therefore, in the present case, detecting the antibody 2 weeks after an immunization with the antigen is reasonable (16). Previous to this present case report, there were reports of two MOGAD cases, one involving a 19-year-old male and another a 47-year-old female, both developing 2 weeks after revaccination against diphtheria, tetanus, pertussis, polio, and influenza. In these two cases, both patients had recurrent episodes of demyelinating diseases with frequent relapses that were refractory to treatment (17). Another reported case involved a 37-year-old female, who had transiently tested positive for an MOG antibody with monophasic symptoms of myelitis a few weeks before receiving measles, mumps, rubella, and tetanus vaccinations (18). Therefore, it is possible that MOG antibodies may be produced by several kinds of vaccines, and some vaccines may cause a vaccine-induced unveiling of multiphasic disease or a transient induction of antibody production in monophasic disease.

Including our case, during the current COVID-19 pandemic, there have been three cases of MOGAD occurring after SARS-CoV-2 vaccinations (19, 20). In these cases, the patients developed their neurological symptoms at various times throughout their vaccinations, from 1 to 2 weeks after the first or second vaccine doses (Table 1). In a systematic review analysis of 32 cases of CNS demyelinating diseases after COVID-19 vaccinations, it was reported that 71.8% of CNS demyelinating diseases occurred after the first dose of any of the COVID-19 vaccines. Furthermore, the interval from vaccination to disease onset was usually 3 weeks, but this time interval was dependent on the existence of preexisting conditions in each case. Of these 32 cases, there were 17 cases involving preexisting immune-mediated diseases, including MS (*n* = 7), a clinically isolated syndrome suggestive of MS (*n* = 1), transverse myelitis (*n* = 1), recurrent neurologic diseases (*n* = 2), and other immune or autoimmune diseases (*n* = 6). In three postvaccination MOGAD cases, neurological symptoms were relatively mild and responded well to intravenous prednisolone treatment. Furthermore, all these cases were monophasic without recurrence, although they were observed carefully for the appearance of oligoclonal bands in two cases and continuous MOG-antibody positivity in our case. Especially, as MOG-IgG was positive but the antibody titer was gradually declining, we should observe the clinical relapse and check the positivity of MOG-IgG carefully. A recent report suggested that the risk of transverse myelitis associated with COVID-19 represents 1.2% of all neurological complications (21). However, the risk of developing demyelinating disease following vaccination for COVID-19 is far less than the risk of contracting COVID-19. We therefore do not hesitate to recommend receiving COVID-19 vaccinations in this report.

**Table 1:** A summary of case reports of MOG-IgG-associated disorders after COVID-19 vaccination.

**References**	**Age**	**Sex**	**Vaccine**	**Interval between vaccination and onset**	**Symptoms**	**MRI findings**	**CSF cell count (/μl)**	**CSF Protein (mg/dl)**	**OCBs**	**Treatment**	**Outcome**
Mumoli et al. (19)	43	Male	ChAdOx1 nCOV-19	7 days after first dose	Bilateral lower limbs weakness urinary retention	Multiple brain white matter lesion and LETM	43	40.6	(+)	IVMP	Mostly recovered
Dams et al. (20)	59	Male	ChAdOx1 nCOV-19	14 days after first dose	paresthesia, gait disturbance, urinary and rectal dysfunction	LETM	110	n.d.	(–)	IVMP and PLEX	Partially recovered
Our case	68	Female	mRNA-1273	14 days after second dose	Paresthesia on her right V2 and V3 area	Cerebellar peduncle lesion	0	32	(+)	IVMP	Partially recovered

*MOGAD, MOG antibody associated disorders; CSF, cerebrospinal fluid; OCB, oligoclonal band; LETM, longitudinally extensive transverse myelitis; n.d., not described; IVMP, intravenous methylprednisolone; PLEX, plasma exchange*.

Vaccines may trigger a preexisting latent autoimmune disease as was seen in a previous case of neuromyelitis optica spectrum disorder (NMOSD) (22) involving a patient that was found to be seropositive for the AQP4-IgG over 10 years before the onset of NMOSD. There have been new reports of onset cases of MS and NMOSD in addition to prediagnosed cases with vaccine-induced recurrence in MS (7). In such cases, the mRNA vaccination may cause the activation of nonspecific or specific cellular immunity and elevated cytokines, which could induce the breakdown of the blood brain barrier leading to the entry of antibodies and unveil the potential for MOG-IgG pathogenesis (23).

Although the present case involved a rare condition of MOGAD after the COVID-19 mRNA vaccination, detailed studies of additional clinical cases are necessary to determine the causation and the risk of developing MOGAD. It is premature to discuss the causal relationship of demyelinating disease MOGAD following COVID-19 vaccination. We have revealed, however, MOGAD's rarity and relatively benign course after vaccination compared with refractory cases of COVID-19-related ADEM and AHLE. It is important to note that the public should not avoid being vaccinated for COVID-19 for fear of developing MOGAD.

## Data Availability Statement

The raw data supporting the conclusions of this article will be made available by the authors, without undue reservation.

## Ethics Statement

The studies involving human participants were reviewed and approved by the Ethical Committee of Tohoku University Hospital. The patients/participants provided their written informed consent to participate in this study. Written informed consent was obtained from the individual(s) and/or minor(s)' legal guardian/next of kin for the publication of any potentially identifiable images or data included in this article.

## Author Contributions

YM, AO, and TM contributed to conception and design of the study. YM wrote the first draft of the manuscript. TM wrote the additional sections of the manuscript. AO, TK, KI, and HW contributed to the clinical analysis. KK and YT assessed the patient. TT did the diagnostic test. TM and MA supervised this report. All authors contributed to the manuscript's revision, and read and approved the submitted version.

## Funding

This work was supported by JSPS KAKENHI grant no. 19K07953 and the MEXT/JSPS WISE Program: Advanced Graduate Program for Future Medicine and Health Care, Tohoku University.

## Conflict of Interest

The authors declare that the research was conducted in the absence of any commercial or financial relationships that could be construed as a potential conflict of interest.

## Publisher's Note

All claims expressed in this article are solely those of the authors and do not necessarily represent those of their affiliated organizations, or those of the publisher, the editors and the reviewers. Any product that may be evaluated in this article, or claim that may be made by its manufacturer, is not guaranteed or endorsed by the publisher.
